# Long-term memory interference is resolved via repulsion and precision along diagnostic memory dimensions

**DOI:** 10.3758/s13423-022-02082-4

**Published:** 2022-04-05

**Authors:** Maxwell L. Drascher, Brice A. Kuhl

**Affiliations:** 1 Department of Psychology, University of Oregon, Eugene, OR USA; 2 Institute of Neuroscience, University of Oregon, Eugene, OR USA

**Keywords:** Episodic memory and recall, Forgetting; Interference, Memory distortion

## Abstract

When memories share similar features, this can lead to interference, and ultimately forgetting. With experience, however, interference can be resolved. This raises the important question of how memories change, with experience, to minimize interference. Intuitively, interference might be minimized by increasing the precision and accuracy of memories. However, recent evidence suggests a potentially adaptive role for memory distortions. Namely, similarity can trigger exaggerations of subtle differences between memories (repulsion). Here, we tested whether repulsion specifically occurs on feature dimensions along which memories compete and whether repulsion is predictive of reduced memory interference. To test these ideas, we developed synthetic faces in a two-dimensional face space (affect and gender). This allowed us to precisely manipulate similarity between faces and the feature dimension along which faces differed. In three experiments, participants learned to associate faces with unique cue words. Associative memory tests confirmed that when faces were similar (face pairmates), this produced interference. Using a continuous face reconstruction task, we found two changes in face memory that preferentially occurred along the feature dimension that was “diagnostic” of the difference between face pairmates: (1) there was a bias to remember pairmates with exaggerated differences (repulsion) and (2) there was an increase in the precision of feature memory. Critically, repulsion and precision were each associated with reduced associative memory interference, but these were statistically dissociable contributions. Collectively, our findings reveal that similarity between memories triggers dissociable, experience-dependent changes that serve an adaptive role in reducing interference.

**Supplementary Information:**

The online version contains supplementary material available at 10.3758/s13423-022-02082-4.

## Introduction

When episodic memories are similar, this can lead to interference and forgetting. A critical point of emphasis in theories of episodic memory has been to not only characterize the contexts and situations in which interference occurs, but to consider the mechanisms that resolve interference (Anderson, 2003; Anderson et al., 1994; Anderson & Spellman, 1995; Crowder, 2014; Fawcett & Hulbert, 2020; Smith & Hunt, 2000). To the extent that similarity is a root cause of interference, one potentially powerful way to reduce interference is to accentuate subtle differences between memories (Hulbert & Norman, 2015; Smith & Hunt, 2000). However, there is surprisingly little evidence characterizing whether or how the contents of episodic memories change as an adaptive response to interference.

One way to accentuate differences between similar memories is by increasing memory *precision*. For example, if two students look similar, more precise memories for the features of those students’ faces (e.g., their specific eye colors) should render those memories more distinct. This concept is similar to the idea from perceptual learning that stimulus dimensions are “stretched” to allow more fine-grained perceptual discriminations (Goldstone, 1998; Nosofsky, 1986). Analogously, increasing memory precision should expand the space between similar memories, thereby reducing interference.

An alternative, though not mutually exclusive, possibility is that differences between similar events are accentuated by *misremembering* event features as being more different than they actually were. For example, a pair of recent studies demonstrated that when otherwise identical objects were associated with slightly different colors, the color difference between those objects was systematically exaggerated in memory (Chanales et al., 2021; Zhao et al., 2021). Critically, this memory *repulsion* only emerged with extensive practice and coincided with reductions in interference-related memory errors. In fact, during early stages of learning, there was an “attraction” in color memory (Chanales et al., 2021). Notably, repulsion-like biases have also been observed in working memory (Bae & Luck, 2017; Chunharas et al., 2018; Chunharas et al., 2019; Golomb, 2015) and visual attention (Chen et al., 2019; Won et al., 2020; Yu & Geng, 2019).

To the extent that episodic memory interference triggers changes in precision or bias, these changes should be most likely to occur (or most beneficial) along feature dimensions that are *diagnostic* of differences between similar memories. For example, if two students have identical hair color but slightly different eye color, then eye color would represent a diagnostic feature dimension. Targeted changes in discrimination accuracy along diagnostic feature dimensions have been observed during category learning (Goldstone & Steyvers, 2001; Kruschke, 1996; Theves et al., 2020) and in working memory (Chunharas et al., 2018). Computational models of episodic memory interference have proposed that episodic memory representations also undergo targeted changes that specifically exaggerate differences between similar memories (Hulbert & Norman, 2015), but empirical support for this proposal remains limited.

While precision and bias may both contribute to the resolution of memory interference, they are orthogonal constructs. Whereas precision refers to a reduction in memory variability, bias refers to a shift in a memory distribution. However, both measures require that memory be expressed using continuous values. Additionally, calculating precision requires that individual memories be sampled multiple times (to observe variability in the response). Despite recent progress towards utilizing continuous feature measures in episodic memory research (e.g., Berens et al., 2020; Brady et al., 2013; Cooper et al., 2019; Cooper & Ritchey, 2019; Harlow & Donaldson, 2013; Harlow & Yonelinas, 2016; Nilakantan et al., 2017; Nilakantan et al., 2018; Rhodes et al., 2020; Richter et al., 2016), prior studies have not specifically compared the relative contributions of precision and bias to the resolution of episodic memory interference.

Here, using multi-dimensional stimuli (faces), we tested whether similarity between stimuli induces adaptive changes in episodic feature memory (precision and/or bias) along diagnostic versus non-diagnostic feature dimensions. We developed a set of synthetic face stimuli that were manipulated on subjectively relevant dimensions (Oosterhof & Todorov, 2008) as well as a behavioral face reconstruction task that allowed participants to express face memory by actively adjusting the synthetic faces. We used this innovative methodology across three experiments (including a preregistered third experiment) that each included a simple learning paradigm in which participants studied associations between faces and cue words (professions). Critically, most of the faces had a competitive *pairmate* that differed only on a counterbalanced diagnostic dimension (affect or gender). After extensive study and retrieval practice, we probed participants’ memories for both feature dimensions simultaneously. Our central hypothesis was that competition would yield adaptive changes along the diagnostic feature dimension. Specifically, we predicted that memory for diagnostic features would be biased to exaggerate differences between similar memories (repulsion) and that repulsion would be associated with lower memory interference. We also predicted greater precision for diagnostic features and, importantly, tested whether repulsion and precision were independently predictive of memory interference.

## Methods

We conducted three experiments with the same core experimental design and procedure. The only differences across the experiments were (1) the similarity of competitive pairmates increased very slightly from Experiments 1 to 2 to 3, and (2) the minimum number of learning rounds was increased from Experiment 1 to Experiments 2 and 3 to account for the greater similarity/difficulty. Analyses and predictions for Experiment 3 were preregistered (https://osf.io/s2gnq) after analyzing data from Experiments 1 and 2. Thus, analyses are first reported for Experiments 1 and 2, and then, separately, for Experiment 3 (to test for replication). Exploratory analyses that combined data across experiments are also reported.

### Participants

Participants were undergraduate students from the University of Oregon who received course credit for participation. A total of 40 participants were recruited for Experiment 1. Four participants were excluded from analyses due to technical/procedural errors (see preregistration for full exclusion criteria: https://osf.io/s2gnq), resulting in a sample of 36 participants (*M*_*age*_ = 19.11 ± 1.65 years, range 18–25 years; 25 females). We sought a similar sample size in Experiment 2 and recruited 41 participants (*M*_*age*_ = 20.49 ± 2.47 years, range 18–28 years; 28 females); no participants were excluded for technical/procedural errors. Based on the effect sizes in Experiments 1 and 2 and corresponding power analyses, we recruited a sample 60 participants for a preregistered Experiment 3 (see https://osf.io/s2gnq). Three participants were excluded for technical/procedural errors, resulting in a sample of 57 participants (*M*_*age*_ = 19.00 ± 2.41 years, range 18–22 years; 40 females). Each experiment involved a single session for each participant that lasted 90–120 min. Informed consent was obtained in accordance with procedures approved by the University of Oregon Institutional Review Board. All participants who were not excluded due to technical/procedural errors were included in our analyses of the associative memory test performance (see *Procedure*). Inclusion in all subsequent analyses was based on a set of performance-based exclusion criteria (see *Performance-based exclusion criteria*).

### Materials

#### Cue words

For each participant and each experiment, the same set of 12 cue words was used (farmer, dentist, lawyer, teacher, chef, tailor, plumber, actor, artist, surgeon, judge, barber). Each cue word was assigned to a unique face, with the assignment randomized for each participant. All of the cue words referred to professions, consisted of one or two syllables, and were displayed in white with all capital letters.

#### Faces

Face images appeared in color with a uniform ellipse shape with a horizontal radius of 81 pixels and a vertical radius of 120 pixels. For all experiments, face images were generated from a set of eight *base faces*. The base faces were derived from a separate experimental procedure in which participants sorted a corpus of 1,008 faces into “families” based on subjective assessment of the likelihood that faces were genetically related. Clustering algorithms were applied to the sorting responses to identify distinct clusters (families). Each of the eight base faces represents the mean face from a cluster, normalized for features not relevant to the grouping (see https://osf.io/6cew9/ for full details of stimulus-generation methods). Critically, because of the way in which the eight base faces were generated, the base faces were distinct from each other according to characteristics that were orthogonal to the dimensions of affect and gender (which were the dimensions manipulated in the current experiments).

For each participant in each experiment, half of the base faces (four) were assigned to a *competitive condition* and half (four) were assigned to a *non-competitive condition*. The assignment of base faces to conditions was randomized for each participant. Base faces were manipulated along two dimensions – affect and gender – in order to generate the specific faces that participants studied (*studied faces*). For the four base faces assigned to the competitive condition, we created pairmates by generating two studied faces from each base face, with the common base being the source of competition. For the four faces assigned to the non-competitive condition, each base face was manipulated to generate a single studied face. Thus, a total of 12 studied faces were generated and used for each experiment.

For each experiment, each studied face was manipulated to fall into one of four locations in a two x two (affect x gender) space. That is, within each experiment, each studied face had one of two affect values and one of two gender values. To manipulate these dimensions, we collected subjective affect and gender ratings for all of the 1,008 faces in the corpus (see https://osf.io/znc58/) and then used regression analyses to learn the mapping between the gender and affect ratings and face image parameters (739 parameters in total) derived from an Active Appearance Model (AAM) (Chang & Tsao, 2017; Cootes et al., 2001; Edwards et al., 1998). Thus, the regression weights allowed for different affect and gender values to be translated to the 739-parameter feature space to manipulate the base faces. In order to maximize the independence of the affect and gender dimensions, for each of the AAM parameters, the dimension (affect or gender) with the highest magnitude regression weight was retained and the regression weight for the other dimension was set to 0. Thus, each face dimension (affect, gender) was associated with a distinct set of AAM parameters.

**Fig. 1:**
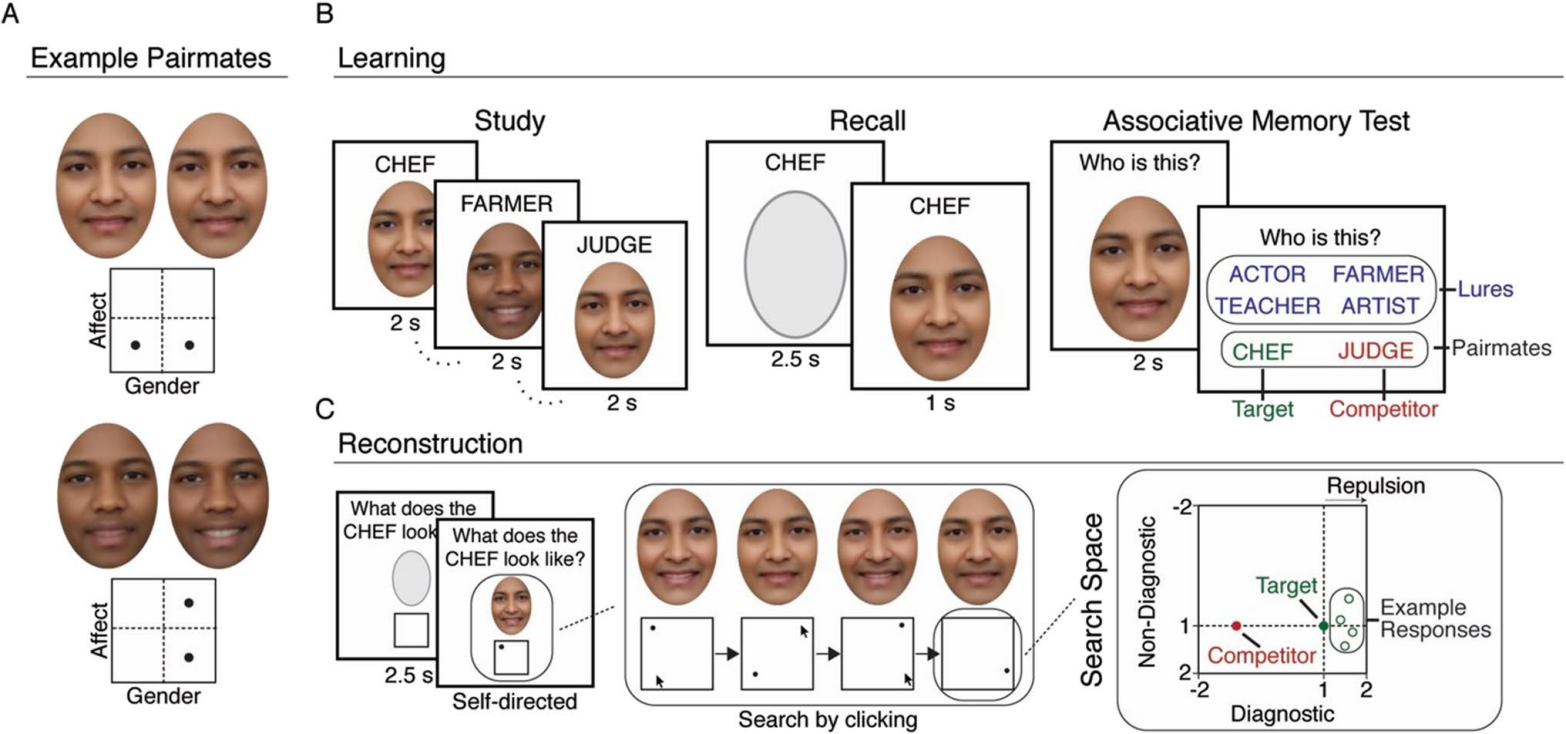
Experimental paradigm and design. **a** Examples of competitive pairmates from Experiment 1, with the location of the faces in affect-gender space shown below. Top: Example of pairmates matched on affect (non-diagnostic dimension) but differing slightly on gender (diagnostic dimension). Bottom: Example of pairmates matched on gender (non-diagnostic dimension) but differing slightly on affect (diagnostic dimension). **b** Learning phase. Each round of the learning phase (up to 12 rounds total) consisted of three tasks. During study, participants viewed and studied associations between cue words and faces. During recall, participants viewed a cue word and were instructed to recall the corresponding face as vividly as possible; the correct face image then appeared. During the associative memory test, participants attempted to match each face image with its corresponding cue word, selected from a set of six options: target, competitor (the cue word of the pairmate face), and four lures (cues from other faces). **c** Face reconstruction task. Left: Participants were first shown a cue and instructed to visualize the corresponding face. Then, an altered version of that face appeared (shifted a random amount on the affect and gender dimensions). Center: Participants used mouse clicks in a two-dimensional box to search the affect-gender space until the reconstructed face matched their memory for the target. Right: Schematic of the search space showing the true location of the target (green dot) and competitor (red dot). Example reconstruction responses (open green dots) demonstrate our predictions: A bias away from the competitor (repulsion) on the diagnostic dimension and lower variability (greater precision) along the diagnostic compared to the non-diagnostic dimension

For the non-competitive condition, the four studied faces corresponded to the four locations in affect-gender space (one face per location), with the assignment of base faces to locations randomly determined for each participant. For the competitive condition, the eight studied faces again corresponded to the four locations in affect-gender space (two faces per location), with the assignment of base faces to locations randomly determined for each participant. Critically, the eight faces in the competitive condition included four sets of pairmates. For two of those sets, the pairmates within each set differed on affect and were matched on gender (i.e., diagnostic dimension = affect, non-diagnostic dimension = gender). For the other two sets, the pairmates differed on gender and were matched on affect (i.e., diagnostic dimension = gender, non-diagnostic dimension = affect) (see Fig. 1a). For the sets of pairmates that shared the same diagnostic dimension, each set corresponded to a different value on the non-diagnostic dimension, but the pairmates within each set had the same value on the non-diagnostic dimension. For example, for the two sets of pairmates for which gender was the diagnostic dimension, each set of pairmates would have a different value on the affect dimension, but the pairmates within each set would have the same value on the affect dimension.

For Experiment 1, the difference between competitive faces along the diagnostic dimension was determined based on subjective assessment of the authors and initial pilot data. The goal was for the differences to be very subtle, yet learnable (see Fig. 1a for examples). *Note:* The units for these differences were not meaningful and are therefore not reported. For Experiment 2, the difference between competitive pairs was reduced by 25% relative to Experiment 1 in order to slightly increase the difficulty/interference. This was motivated by evidence that repulsion is more likely to occur when discrimination is relatively more difficult (Chanales et al., 2021). For Experiment 3, the difference between competitive pairs on the gender dimension was the same as in experiment 2, but the difference on the affect dimension was reduced by 50% relative to Experiment 1. This was motivated by evidence, from Experiment 2, that interference was somewhat lower along the affect dimension compared to the gender dimension. Note that since the differences between competitive pairs in Experiment 1 were quite small to begin with, the changes across experiments were subtle. For additional consideration of differences between affect versus gender across experiments, see Fig. S1 in the Online Supplemental Material (OSM).

Within each experiment, the difference between competing faces (pairmates) on the diagnostic dimension is described in *relative terms* (scaled units), with each face being 1 unit from the center of face space and, therefore, 2 units from each other. All faces were also exactly 1 unit away from the affect and gender borders in the response window (see *Reconstruction phase*, below). Analyses of face memory from the reconstruction phase were performed based on the distance, in units, between participants’ responses and the actual location of the studied phases.

### Procedure

Each experiment consisted of two main phases: a learning phase and a reconstruction phase. The purpose of the learning phase was for participants to extensively study and practice remembering the cue-face associations. The reconstruction phase served as the critical memory test for measuring bias and precision in face memory. All experiments were run in Matlab, using the Psychophysics Toolbox extensions (Brainard, 1997; Kleiner et al., 2007; Pelli, 1997). All phases of the experiment had a gray background.

#### Learning phase

The learning phase consisted of up to 12 rounds, with each round split into two sub-rounds. Each sub-round included three blocks corresponding to the following experimental tasks, in the following order: study, recall, and associative memory test (Fig. 1b), with the exception that rounds one and two did not include the recall task. For each participant and each round of the learning phase, the 12 associations were randomly split into two groups of six associations each (four competitive, two non-competitive), with each group of six associations assigned to a separate sub-round. In other words, in each round of the learning phase, half of the associations went through study/recall/associative memory test and then the other half of the associations went through study/recall/associative memory test (with the exception, as noted above, that rounds one and two did not include the recall task). The rationale for splitting the associations into two sub-rounds was to facilitate learning by reducing the amount of information per block.

In the study task, participants viewed and studied the cue-face pairings. On each trial (2,000 ms), a cue appeared directly above a face image. In between trials, there was a fixation cross for 200 ms. Participants were instructed to study the cue-face pairings; no response was made. In the recall task, participants attempted to recall the face associated with each cue. On each trial, a cue was presented above a blank ellipse (representing the to-be-recalled face) for 2,500 ms. Participants were instructed to recall the associated face image as vividly as possible. Although no response was made, the correct face would then appear below the cue for 1,000 ms as a way of providing feedback. In between trials, there was a 200-ms fixation cross. In the associative memory test, participants attempted to match face images with corresponding cue words. On each trial, a face image was presented for 2,000 ms and was then replaced by a set of six different cue words displayed in the bottom half of the screen (three cues in each of two rows with the position randomly determined for each trial). The cue words included all of the cues from the current sub-round. For faces in the competitive condition, the set of cues included the correct answer (target), the cue that had been paired with the current face’s pairmate (interference error), and four cues that had been paired with the other, unrelated faces (lures). For faces in the non-competitive condition, the set of cues included the correct answer (target) and five cues that had been paired with unrelated faces (lures). Participants made responses by clicking on the cue word with the mouse. After each response was registered, feedback indicated whether the response was correct (“Correct!”; 500 ms) or incorrect with the correct cue indicated (e.g., “Incorrect. This is the BARBER.”; 2,000 ms).

During the first two rounds of the learning phase, each study block presented each cue-face association three times. In subsequent rounds, each association was studied once per block. As noted above, there was no recall task in the first two rounds of the learning phase. In subsequent rounds, each association was recalled twice per recall block. Across all rounds of the learning phase, each association was tested three times per associative test block. For each task block (study/recall/associative test), the order in which each association was presented/tested was pseudo-randomly determined, with the following constraints: (1) all of the associations in each block were studied/presented once before any were repeated, (2) a given association was never presented/tested consecutively, (3) competing associations (face pairmates) were never presented/tested in consecutive trials. These constraints helped ensure that any comparisons between stimuli/associations were memory-based.

In Experiment 1, participants repeated the learning phase for at least nine rounds and until they reached 100% accuracy on the associative memory test, up to a maximum of 12 rounds. Most participants had reached perfect accuracy after nine rounds (24/36), and nearly all did so after ten rounds (31/36). Only two participants went through all 12 rounds, with one achieving perfect performance and the other being removed for continued poor performance (see below for performance-based exclusion criteria). In Experiments 2 and 3, all participants completed 12 rounds of the learning phase regardless of associative memory test performance. For each experiment, participants were given the opportunity to take a break after every two rounds, with the length of the break determined by the participant. Participants were instructed to press the space bar when ready to proceed.

#### Reconstruction phase

After the learning phase, participants’ memories for the features of the faces were probed with a surprise reconstruction task (Fig. 1c). On each trial in the reconstruction task, participants were first shown a cue (e.g., “What does the BARBER look like?”) above a blank ellipse for 2,500 ms and were instructed to bring the target face to mind. Next, an altered version of the target face appeared in the ellipse with a response box beneath the face representing the search space (see *Reconstruction search space*, below, for details). Participants used a mouse to click through the box; the face image above the box changed according to the location of each mouse click in the box. Although participants were not explicitly made aware of this, the box represented a two-dimensional affect-gender space. Participants were instructed to continue searching (clicking through the box) until the face matched their memory for the target face. Participants finalized their response by pressing the space bar. There was no limit on the response time. A fixation cross appeared for 200 ms between trials. Each of the 12 studied faces was probed (reconstructed) a total of four times in the reconstruction phase (48 trials total). The rationale for probing faces multiple times was so that the precision (variability) of reconstructions for each face could be measured. Faces were reconstructed in a pseudo-random block order. In each of four consecutive blocks (with no break or demarcation between blocks), each of the 12 faces was reconstructed once. As in the learning phase, the same face was never tested consecutively and pairmate faces were never tested in consecutive trials. After the reconstruction phase, there was a short phase where participants were prompted to provide a rating on a 9-point scale for both affect and gender for each stimulus. Results from this task (which was only included for validation) are not described here.

#### Reconstruction search space

In the reconstruction task, the altered face presented on each trial was derived from the same base face as the target face, but the affect and gender values were randomly selected from a range of possible values. This range of possible values corresponded to the size of the two-dimensional search space (i.e., the size of the response box). Importantly, the range of the search space and the center of the search space were identical across all trials, but the mapping of the dimensions to the x and y axes (e.g., x axis = affect, y axis = gender) and the direction/orientation of the axes (e.g., left = low, right = high) were randomly varied for each trial so that participants would not learn to associate a given face with a fixed spatial position in the response box. For each experiment, the size of the search space relative to the distance between pairmate faces was identical. That is, for each experiment the height and width of the search space was exactly twice the distance between pairmate faces on the diagnostic dimension. Thus, with pairmate faces 2 units apart (in our standardized units), the height and width of the search space was 4 units. For each trial, the location of the correct answer (target face) and the location of the pairmate face (for faces in the competitive condition) always corresponded to one of four possible locations (the center of each quadrant) with all four of those locations contained in the search space (see Fig. 1a).

### Analysis methods

#### Performance-based exclusion criteria

For analyses that involved the reconstruction task data, we excluded a small number of participants based on performance during rounds 9–12 of the associative memory test. Participants were excluded if (a) their error rate for non-competitive trials was greater than 20% for any of these rounds or (b) they selected the lure faces on greater than 20% of the competitive trials for any of these rounds. Based on these criteria, one participant was excluded from analysis of the reconstruction task data in Experiment 1 (yielding *N* = 35), four were excluded from Experiment 2 (yielding *N* = 37), and eight were excluded from Experiment 3 (yielding *N* = 49) (see https://osf.io/dj6q2/ for other exclusion criteria that were established but did not apply). The rationale for having a high threshold for inclusion of participants in the reconstruction task analysis was to minimize cases where participants reconstructed an entirely wrong face and to instead focus on bias/precision in otherwise correctly remembered faces.

#### Measuring associative memory

As noted above, the associative memory test was used to confirm that participants achieved high accuracy in associating cues with faces. The associative memory test also allowed for a manipulation check of whether the competitive condition induced interference (lower associative memory accuracy) compared to the non-competitive condition. Data from the associative memory test was first analyzed in terms of accuracy on competitive compared to non-competitive trials. We ran a separate repeated-measures ANOVA for each experiment with factors of condition (competitive, non-competitive) and learning round (1–9 for Experiment 1, 1–12 for Experiments 2 and 3). For competitive trials, we also separated errors according to whether they were attributable to competition (interference error) or not (lures). If errors were random, interference errors would occur on one-fifth (20%) of the error trials. To test whether interference errors occurred at above chance levels, we therefore ran one-sample *t*-tests for each experiment, comparing the mean percentage of interference errors (across all learning rounds) to 20%.

#### Measuring bias

As described above, on each trial in the reconstruction task the target face was located in one of four locations (the center of the four quadrants). Thus, for both the x and y axes of the search space, the target was half-way between the center and the border of the search space (Fig. 1a). To measure for potential bias, for each experiment all responses were aligned onto a common axis and rescaled onto a common scale, separately for each feature dimension (affect, gender). For the rescaled data, the range of possible responses for each dimension was -2 to 2, with 0 being the center of the face space (i.e., the center of the search space). For the competitive condition, the location of the target face on the diagnostic dimension = 1 and the location of the pairmate face = -1 (Fig. 1c). Thus, a bias *away* from the pairmate face would be represented by values greater than 1, whereas a bias *toward* the pairmate face (or toward the center of face space) would be represented by values lower than 1. For the non-diagnostic dimension, the location of the target face *and* the pairmate face = 1. Although faces from the non-competitive condition were included in the reconstruction task, bias was not measured for these faces because the distinction between diagnostic versus non-diagnostic dimensions did not exist. Rather, non-competitive faces were of critical importance in the associative memory test, where they served to establish an overall memory interference effect.

It is important to note that, for the reconstruction task, the response range on each trial was asymmetrically distributed around the target. If the response range had been symmetrically distributed around the target, then the correct response on each trial would have, by definition, been the center of the search space – which likely would have led participants to learn to simply respond in the center. However, the drawback of the approach we used is that, for the diagnostic dimension in the competitive condition, there was more opportunity to respond *toward* the pairmate face (values between -2 and 1) than *away from* the pairmate face (values of 1 to 2). Of course, this asymmetry works *against* our predicted effect of repulsion (values greater than 1). Nonetheless, in order to account for the asymmetrically restricted response range, we estimated the true mean by fitting truncated normal distributions to the data. For each participant, separate models were run for the diagnostic and non-diagnostic dimensions, with each model pooling data across faces and feature dimensions (affect, gender) in order to include a sufficient number of data points. Thus, each model included 32 data points (eight faces in the competitive condition × four reconstruction trials per face). Maximum-likelihood estimation was used to find the mean and standard deviation of a truncated normal distribution that best fit the data. The distributions were modelled using the truncnorm and MASS packages in R. We constrained the search space of the mean to a range of plausible values evenly balanced on either side of the target (± 1 unit) and constrained the standard deviation to be a maximum of 1 and a minimum of .1. Although we view the modelled means as a better estimate of the true means, there are some sources of variance that the models do not account for. For example, the models do not account for potentially unique distributions for each feature dimension and/or stimulus. Furthermore, there is evidence that there may be inherent, global biases in how face features are later recalled (Bülthoff & Zhao, 2020; Won et al., 2020). Critically, however, any global biases would equally influence the diagnostic and non-diagnostic dimensions. Therefore, our analysis primarily focused on *differences* in modeled means for the diagnostic versus non-diagnostic dimensions.

#### Measuring precision

In order to measure the precision with which diagnostic and non-diagnostic features were remembered *for each face*, we calculated the standard deviation of responses across the four reconstruction trials for each face, separately for the diagnostic and non-diagnostic feature dimensions. We then computed the mean of these standard deviation values for each participant, separately for the diagnostic and non-diagnostic dimensions.

#### Measuring the relationship between reconstruction bias and associative interference

In order to determine whether bias on the diagnostic feature dimension plays an adaptive role in reducing memory interference, we ran a series of mixed-effects models that focused on the relationship between bias measured during the reconstruction task and accuracy on the associative memory test (averaged across the last four rounds in order to capture the end state of learning). Although this analysis was performed at the level of individual items (faces), the accuracy value for each face was defined as the average accuracy for that face and its pairmate. As such, both pairmates with each set had the same accuracy value. The rationale for averaging accuracy across pairmates was that if, for example, participants associate two competing faces (pairmates) with the same cue word (profession), rather than treating one of these associations as “correct” and the other as “incorrect,” it is more appropriate for the error to be shared across the two faces.

For the analyses relating reconstruction bias to associative memory accuracy, we excluded participants who had perfect accuracy, across all trials, on the final four rounds of the associative memory test. The rationale for this exclusion was that, for these participants, there was no variance in associative memory for the model to explain. Additionally, we did not run this analysis for Experiment 1 given the near-ceiling performance on the associative memory test over the last four rounds (11 participants (31%) had 100% accuracy; and the remaining participants had a mean accuracy of 95.96 ± 3.01% with an average SD within a participant of 3.62 ± 1.70). For Experiments 2 and 3 – which used more similar pairmates – associative memory accuracy was lower and, therefore, fewer participants were excluded due to ceiling performance (seven participants (19%) in Exp. 2 and six participants (12%) in Exp. 3; mean accuracy for the remaining participants, Exp. 2: *M* = 92.47 ± 7.58%, Exp. 3: *M* = 93.56 ± 6.26%).

For these models, it was critical to compute reconstruction bias at the level of individual faces. However, the method described above of estimating the average bias for each participant by pooling across trials/faces was not feasible for this analysis given the small number of observations (four trials per face). Thus, for this analysis we simply used the mean of the reconstruction response (across the four trials per face). In order to address the concern that any observed relationship between reconstruction bias and associative memory accuracy might be driven by potential “swap errors,” our preregistered approach was to exclude any individual responses (trials) for which the scaled response was between -2 and 0 and to only retain responses for which the scaled response was between 0 and 2. For the diagnostic dimension, any responses that were closer to the competing pairmate than to the target were therefore excluded. All remaining responses were included in the mean response for each face. While rare, if a face was associated with an excluded response on all four reconstruction trials, that face was entirely excluded from analysis. For Experiment 2, this occurred for a total of four faces distributed across four participants; for Experiment 3, this occurred for a total of six faces distributed across six participants. While this preregistered approach for exclusion of potential swap errors was intended as a conservative approach for eliminating the influence of extreme errors, all of our main results remained significant when no responses were excluded. Additionally, in exploratory analyses that combined data across Experiments 2 and 3, instead of excluding extreme responses altogether, responses between -2 and 0 were capped at a value of 0, which allowed for all trials to be retained in the model, but reduced the influence of extreme responses.

Mixed-effects models were implemented in R using the lme4 package (Bates et al., 2014). Likelihood ratio tests were used to compare models with relevant variables to null models that excluded those variables. In order to account for potential differences related to whether the diagnostic dimension was affect versus gender, all models included this categorical variable as a fixed effect. In order to allow the relationship between reconstruction bias and associative memory accuracy to vary for each participant, we modeled the relationship between bias and associative memory accuracy with random intercepts and random slopes for each participant, where possible. Our preregistered approach to dealing with models that failed to converge or that reached a singular fit was to rerun the same model with the random slope for bias removed (see Barr et al., 2013). While all of our preregistered models did converge, an exploratory model that used the difference in bias on the diagnostic versus non-diagnostic dimension as a predictor failed to converge when a random slope was included; thus, we removed the random slope. Exploratory models that included only unsigned error or precision as predictors (without bias) failed to converge when random slopes were included for these variables; thus, we removed random slopes for these variables. Finally, exploratory models that included bias along with precision and unsigned error as predictors also failed to converge when random slopes were included for all variables; when removing random slopes, we prioritized retaining a random slope for bias, which led to the exclusion of random slopes for precision and unsigned error.

## Results

### Associative memory test

**Fig. 2:**
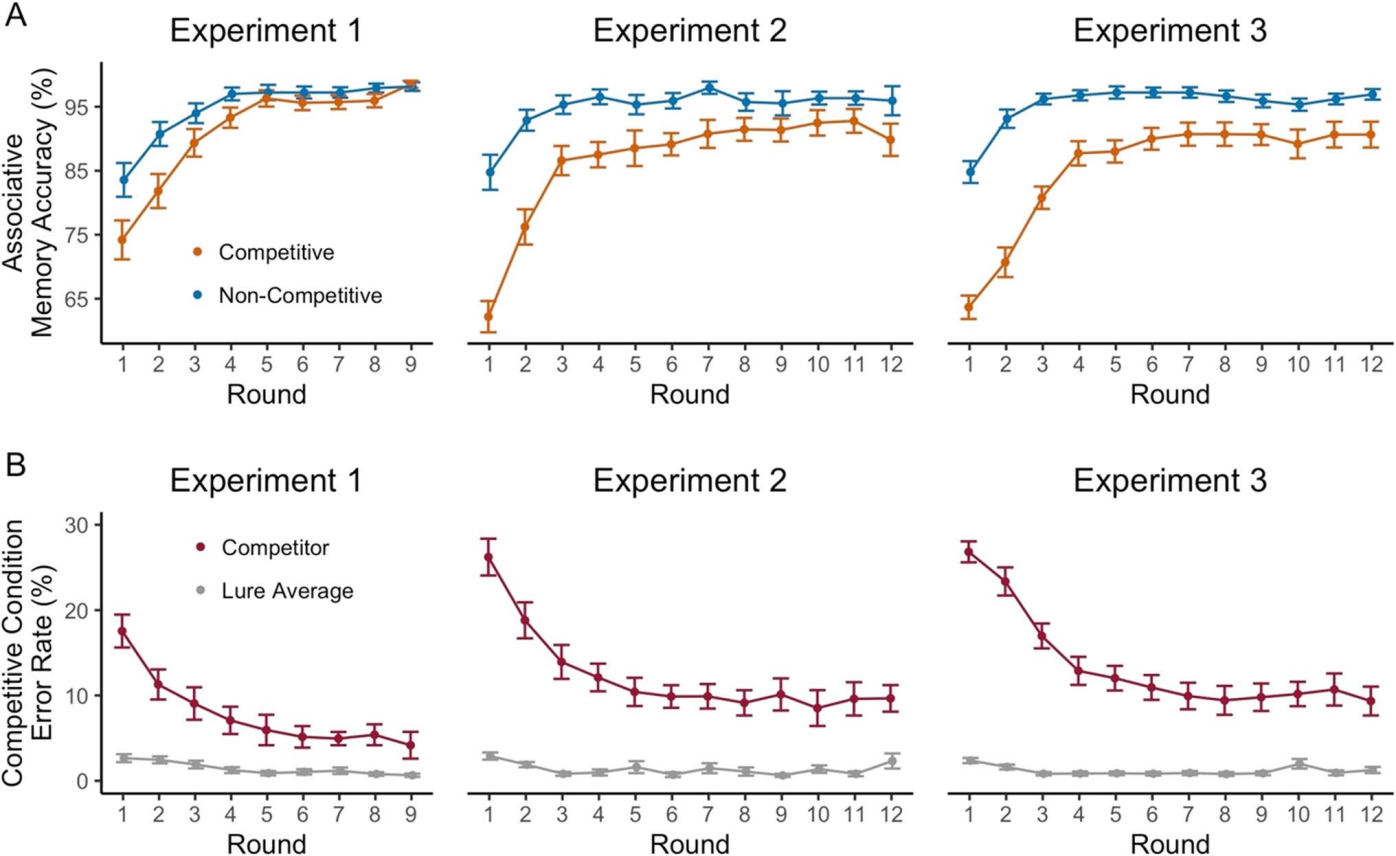
Associative memory test accuracy across learning rounds. **a** Percent correct responses on the associative memory test during each round of the learning phase, separated by the non-competitive (blue) and competitive (orange) conditions and by experiment number. Performance was significantly higher for the non-competitive compared to the competitive condition in each of the three experiments. For accuracy in the competitive condition separated according to whether the diagnostic dimension was affect versus gender, see Fig. S1A (Online Supplemental Material). **b** Error rates for the competitive condition on the associative memory test during each round of the learning phase. Data are separated by error type (competitor: red; lure average: grey) and experiment number. Competitors (the cues associated with the pairmate faces) were selected at a rate that exceeded the average rate of selecting one of four lures. Error bars represent SEM

To test whether associative memory accuracy differed between the competitive and non-competitive conditions, we conducted repeated-measures ANOVAs for each experiment with factors of condition (competitive, non-competitive) and round (Exp. 1: the first nine rounds; Exp. 2 and Exp. 3: 12 rounds). For each experiment, there was a significant main effect of condition (Exp. 1: *F*(1,35) = 26.14, *p* < 0.001, ${\eta}_G^2$ = 0.034; Exp. 2: *F*(1,40) = 67.43, *p* < 0.001, ${\eta}_G^2$ = 0.10; Exp. 3: *F*(1,56) = 88.21, *p* < 0.001, ${\eta}_G^2$ = 0.16), with lower accuracy in the competitive condition (Fig. 2a). To confirm that this difference specifically reflected interference, we considered the types of errors made. For the competitive condition, errors could correspond to selecting the competitor face or one of the four non-competitive lures (Fig. 2b). If errors were random, the competitor would be selected on one-fifth of the error trials. However, combining error trials across rounds, the competitor was selected at above-chance levels (Exp. 1: *M* = 60.18 ± 19.68%, *t*(35) = 12.25, *p* < 0.001, *d* = 2.04; Exp. 2: *M* = 71.29 ± 15.78%, *t*(40) = 20.82, *p* < 0.001, *d* = 3.25; Exp. 3: *M* = 78.63 ± 11.58%, *t*(56) = 38.21, *p* < 0.001, *d* = 5.06), confirming that increased errors in the competitive condition reflected interference from the competitor face.

### Face reconstruction accuracy

**Fig. 3:**
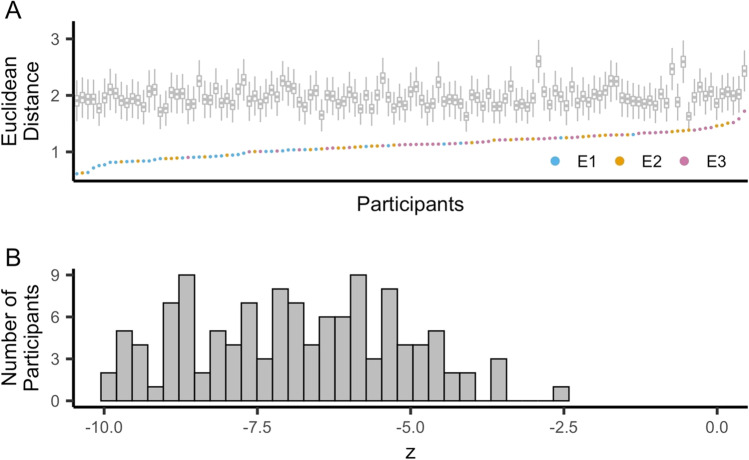
Face reconstruction accuracy. **a** The mean Euclidean distance between the reconstructed location and the target location was significantly lower than chance for every participant as determined by comparing responses to a distribution of shuffled responses (10,000 shuffles per participant). The plot is arranged from participants with the lowest to highest mean Euclidean distance (left to right), with each participant represented by an individual dot (Exp. 1: blue; Exp. 2: orange; Exp. 3: pink). The distribution of shuffled responses for each participant is represented by a boxplot. **b** Histogram of z scores reflecting each participant’s mean Euclidean distance relative to the distribution of shuffled data (*M* = -6.87 ± 1.68, *range* = [-9.97, -2.59]). Lower z scores reflect better performance (lower Euclidean distance)

To test whether face reconstruction accuracy was above chance, we measured the Euclidean distance between each response and the target face location (in the two-dimensional response space; Fig. 1c). For each participant, the mean Euclidean distance between responses and target locations was compared against a permuted distribution (calculated by shuffling responses within participant 10,000 times). Above-chance accuracy (better than 97.5% of the permuted means) was observed for every participant (Fig. 3).

### Face reconstruction bias

**Fig. 4:**
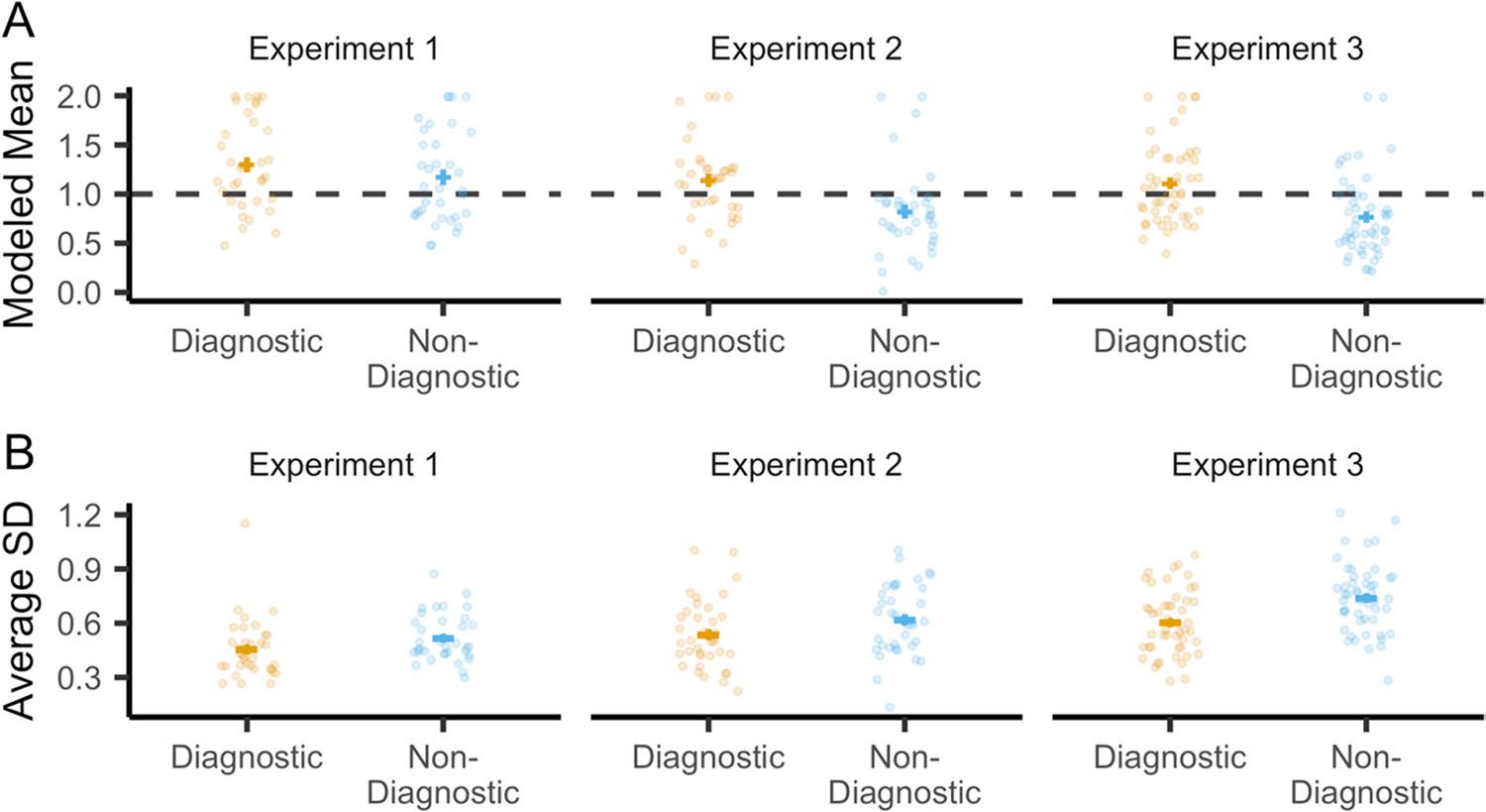
Feature memory from the reconstruction task along the diagnostic and non-diagnostic dimensions. **a** There was greater bias towards repulsion (higher modeled mean response) on the diagnostic (orange) compared to the non-diagnostic (blue) dimension. **b** There was greater precision (lower standard deviation of responses across the four reconstruction trials for each face) on the diagnostic compared to the non-diagnostic dimension. For analyses separated according to whether the diagnostic dimension was affect versus gender, see Fig. S1B and C (Online Supplemental Material, OSM). For analyses comparing the diagnostic and non-diagnostic dimensions with the non-competitive condition, see Fig. S3 (OSM). Error bars represent SEM

To test our critical prediction of repulsion along the diagnostic face dimension, we compared feature bias (see *Methods*) for the diagnostic versus non-diagnostic dimensions in the competitive condition (Fig. 4a). We first tested predictions in Experiments 1 and 2, and then tested for replication in Experiment 3. A repeated-measures ANOVA with factors of dimension (diagnostic, non-diagnostic) and experiment (Exp. 1, Exp. 2) revealed significantly greater bias toward repulsion on the diagnostic dimension (*F*(1,70) = 22.25, *p* < 0.001, ${\eta}_G^2$ = 0.061). There was a trend toward a significant interaction between dimension and experiment (*F*(1,70) = 3.96, *p* = 0.0506, ${\eta}_G^2$ = 0.011), with a relatively weaker effect size in Experiment 1 (*d* = 0.27) than in Experiment 2 (*d* = 0.73). As predicted, Experiment 3 replicated, with a large effect size and preregistered hypothesis, the greater bias toward repulsion on the diagnostic dimension (*t*(48) = 5.87, *p* < 0.001, *d* = 0.83).

Although our preregistered analyses focused on the *comparison* between diagnostic and non-diagnostic dimensions, we also tested whether reconstructions on the diagnostic dimension significantly differed from the veridical location of target faces. Indeed, combining data across all three experiments, the modeled means for the diagnostic dimension were significantly greater than the true value of 1 (*t*(120) = 4.39, *p* < 0.001, *d* = 0.40), reflecting a bias away from the competing face. This effect did not significantly differ across experiments (*F*(2,118) = 2.15, *p* = 0.12, ${\eta}_G^2$ = 0.035). In contrast, on the non-diagnostic dimension there was a small but significant bias toward the center of face space (modeled means < 1; *t*(120) = -2.33, *p* = 0.021, *d* = 0.21). This effect significantly differed across experiments (*F*(2,118) = 9.56, *p* < 0.001, ${\eta}_G^2$ = 0.14). In fact, in Experiment 1 responses were significantly above 1 (*t*(34) = 2.15, *p* = 0.039, *d* = 0.36), and in Experiments 2 and 3 they were significantly below 1 (Exp. 2: *t*(36) = -2.45, *p* = 0.019, *d* = 0.40; Exp. 3: *t*(48) = -3.98, *p* < 0.001, *d* = 0.57). While the absolute values of reconstructed responses should be interpreted with some caution (due to potential global biases), the consistent bias toward repulsion on the diagnostic dimension supports our prediction that competition triggers targeted repulsion on the diagnostic dimension.

### Face reconstruction precision

We next tested whether reconstruction precision differed across diagnostic versus non-diagnostic dimensions (Fig. 4b). We defined precision as the standard deviation across repeated reconstructions of the same face (see *Methods*). For the competitive condition, a repeated-measures ANOVA with factors of dimension (diagnostic, non-diagnostic) and experiment (Exp. 1, Exp. 2) revealed significantly greater precision – i.e., lower reconstruction variability – on the diagnostic dimension (*F*(1,70) = 16.81, *p* < 0.001, ${\eta}_G^2$= 0.044). This effect did not interact with experiment (*F*(1,70) = 0.34, *p* = 0.56, ${\eta}_G^2$ = 0.001). The effect of greater precision on the diagnostic dimension was replicated (consistent with our preregistered prediction) in Experiment 3 (*t*(48) = 5.45, *p* < 0.001, *d* = 0.74).

Although our measure of precision was mathematically independent from our measure of bias, it is notable that these measures were correlated such that faces reconstructed with greater precision also tended to be associated with greater bias (see Fig. S2A, OSM). Importantly, however, the effect of greater precision on the diagnostic versus non-diagnostic dimension remained significant even when high-bias items were excluded from analysis (see Fig. S2B, OSM).

### Relationship between reconstruction bias and associative interference

Finally, we tested our prediction that greater reconstruction bias (repulsion) on the diagnostic dimension is associated with better associative memory test performance (less interference). Due to near-ceiling associative memory performance in Experiment 1 (Fig. 2), we focused on Experiment 2 data. We ran a mixed-effects model that predicted item-level associative memory accuracy with fixed effects of (a) bias on the diagnostic dimension (continuous variable) and (b) whether the diagnostic dimension was affect or gender (categorical variable). Bias was modelled with random intercepts and slopes for each participant. Using a likelihood ratio test, we compared this model to a model without bias. Critically, model fit was significantly better when bias was included (*χ*^2^(1) = 4.67, *p* = 0.031), with bias positively predicting associative memory accuracy (*β*_*bias*_ = 3.58, *SE* = 1.62). As a control, we repeated the same analysis, but with bias on the non-diagnostic dimension; here, bias failed to improve model fit (*χ*^2^(1) = 0.021, *p* = 0.89, *β*_*bias*_ = -0.31, *SE* = 2.14). For Experiment 3, we predicted (using a preregistered analysis) a replication of the relationship between diagnostic dimension bias and associative memory accuracy. We observed a small effect in the predicted direction, but it was not significant (*χ*^2^(1) = 0.24, *p* = 0.63, *β*_*bias*_ = 0.69, *SE* = 1.41).

In our preregistered analysis, we excluded reconstruction responses (trials) that were more similar to the competitor than to the target. The rationale for this was to ensure that extreme responses (potential swap errors) did not have an outsized influence on the model (see *Methods*). However, this approach fully eliminated these trials rather than minimizing their influence. Therefore, as an exploratory analysis, we replaced these extreme reconstruction scores with a value of 0 (equal distance between the target and competitor, see *Methods*). This allowed all trials to be included, but reduced the influence of extreme responses (see Fig. S4 (OSM) for further analysis of what these extreme responses may represent). For this exploratory analysis, we combined data from Experiments 2 and 3, with experiment (Exp. 2, Exp. 3) added as a fixed effect. Compared to a null model, adding bias on the diagnostic dimension significantly improved model fit (*χ*^2^(1) = 15.88, *p* < 0.001), with positive bias (repulsion) predicting higher associative memory accuracy (*β*_*bias*_ = 4.45, *SE* = 1.04). Adding an interaction between experiment and bias, did not improve model fit (*χ*^2^(1) = 1.39, *p* = 0.24, *β*_*exp* × *bias*_ = -2.47, *SE* = 2.08), indicating that the relationship between bias and associative memory did not differ across experiments. Moreover, bias significantly improved model fit when applied to Experiment 3 data alone (*χ*^2^(1) = 3.98, *p* = 0.046, *β*_*bias*_ = 2.45, *SE* = 1.19), confirming that the relationship between bias and associative memory was not driven only by Experiment 2 data. As a control, we ran the same model comparison but with bias on the non-diagnostic dimension as a predictor; there was no significant difference between models (*χ*^2^(1) = 0.14, *p* = 0.71, *β*_*bias*_ = -0.40, *SE* = 1.08). Further, the degree of bias on the diagnostic dimension *relative to* the non-diagnostic dimension (i.e., the bias difference score) also significantly improved model fit compared to a null model without bias,*χ*^2^(1) = 19.87, *p* < 0.001, *β*_*bias*. *diff*_ = 2.71, *SE* = 0.60 (random slopes were excluded due to reaching singularity).

**Fig. 5:**
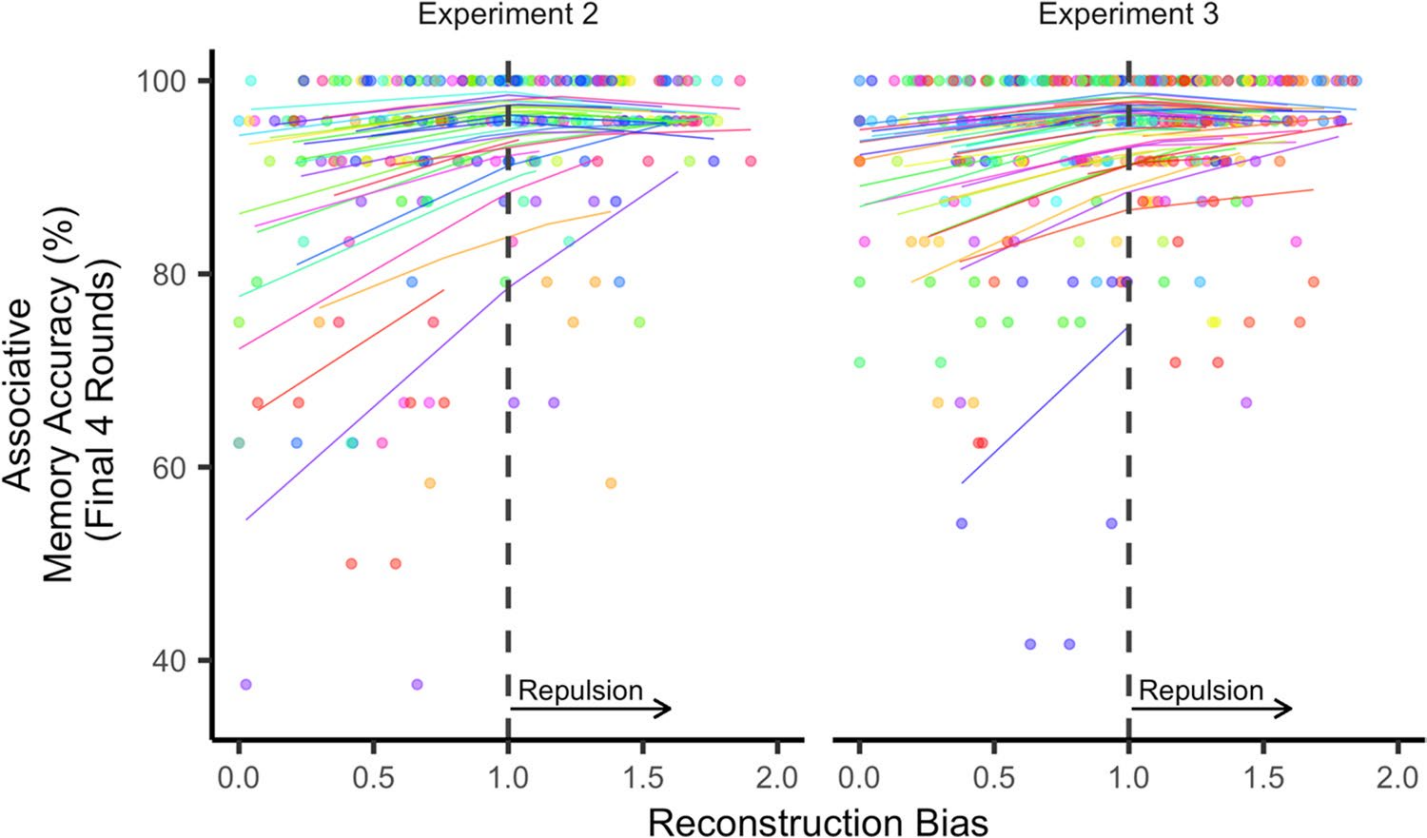
Relationship between reconstruction bias on the diagnostic dimension and associative memory accuracy. For the purpose of visualization, a mixed-effects model was run with mean associative memory accuracy (from the final four rounds of the learning phase) as the dependent variable and with experiment number, unsigned error, and bias included as predictors (gender/affect and precision were excluded). Stronger bias towards repulsion (reconstruction bias values > 1 reflect repulsion) was associated with higher associative memory accuracy (i.e., lower interference). Each dot represents a specific face image, with each participant plotted with a unique color. Each line represents the modelled, participant-specific relationship between reconstruction bias and associative memory accuracy. *Note:* Bends in the lines reflect effects of absolute error

In an additional set of exploratory analyses that again combined data from Experiments 2 and 3 we tested whether reconstruction bias on the diagnostic dimension predicted associative memory accuracy beyond what was predicted by unsigned error (absolute distance from the target on the diagnostic dimension) and precision (on the diagnostic dimension). *Note:* The following analyses did not include random slopes for unsigned error or precision (see *Methods* for rationale). Using hierarchical linear regressions with fixed effects of experiment (Exp. 2, Exp. 3) and feature dimension (whether the diagnostic dimension was affect or gender), model fit was significantly improved, compared to a null model, when unsigned error or precision were added (unsigned error: *χ*^2^(1) = 16.42, *p* < 0.001, *β*_*unsigned*. *error*_ = -5.72, *SE* = 1.40; precision: *χ*^2^(1) = 30.27, *p* < 0.001, *β*_*precision*_ = -5.91, *SE* = 1.06). In other words, lower unsigned error and greater precision were associated with better associative memory. Critically, however, model fit significantly improved when bias was added to a model that already included unsigned error and precision (*χ*^2^(1) = 4.39, *p* = 0.036, *β*_*bias*_ = 2.38, *SE* = 1.11). Thus, bias predicted associative memory accuracy beyond what was explained by precision and unsigned error. Notably, model fit also significantly improved when precision was added to a model that already included unsigned error and bias (*χ*^2^(1) = 26.51, *p* < 0.001, *β*_*prec*_ = -5.64, *SE* = 1.08). Taken together, these exploratory analyses indicate that bias (repulsion) and precision – despite being correlated measures (Fig. S2A, OSM) – were independently predictive of associative memory performance (Fig. 5).

## Discussion

Across three experiments we found that similarity between long-term memories induced adaptive and feature-specific changes to the contents of those memories. We measured these changes using a two-dimensional face space (affect, gender), allowing us to separately measure memory along a dimension that was diagnostic of differences between similar faces and a dimension that was non-diagnostic of differences. We found that memory along diagnostic feature dimensions exhibited two key properties: (1) a systematic bias (repulsion) that exaggerated the difference between similar memories, and (2) greater precision (lower variability). Finally, we found that repulsion and precision were independently predictive of interference-related memory errors.

Although our paradigm was modeled after classic memory interference studies (Anderson, 2003; Anderson et al., 1994; Anderson et al., 2000), the repulsion effect we observed is distinct from classic interference effects. If anything, interference predicts an *attraction* in remembered features. However, an important feature of our design is that face memory was only tested after extensive study and practice (Chanales et al., 2021; Zhao et al., 2021). Indeed, we found that greater repulsion in feature memory was associated with *lower* interference in the associative memory test. While it is important to note that this relationship failed to replicate using our preregistered analysis method in Experiment 3, we view the updated method as a better approach for handling extreme responses, and the relationship we observed generalized across experiments and was independently significant in Experiment 3. The relationship between repulsion and associative memory accuracy is notable when considering that repulsion fundamentally reflects a form of memory *error*. However, the error we observed was not randomly distributed; instead, it was systematically biased away from competing memories, thereby increasing the representational distance between memories. These findings complement evidence of conceptually similar biases in working memory (Bae & Luck, 2017; Chen et al., 2019; Chunharas et al., 2018; Chunharas et al., 2019; Golomb, 2015) and visual attention (Won et al., 2020; Yu & Geng, 2019). The ubiquity of these biases across domains suggests that repulsion is a fundamental, adaptive mechanism for resolving interference.

A central and novel focus of the present study was to compare repulsion along diagnostic versus non-diagnostic feature dimensions. The fact that repulsion was stronger for the diagnostic dimension provides important evidence that memories were not globally exaggerated (relative to the center of face space) in response to competition. Critically, in studies where only one featured dimension is probed (Chanales et al., 2021; Zhao et al., 2021), this interpretation cannot be ruled out. It is also noteworthy that because the mapping between affect and gender and the diagnostic and non-diagnostic dimensions was counterbalanced within participants, our results cannot be explained in terms of a bias along one feature dimension that generalized across all faces, as might occur in category learning (Goldstone, 1998; Goldstone & Steyvers, 2001). Finally, the relationship between repulsion and memory interference was selective to the diagnostic feature dimension, confirming that global biases were not adaptive. Thus, competition triggered targeted and adaptive distortions that preferentially occurred along the dimension that was essential for discrimination. These findings provide novel support for computational models of memory interference that propose targeted, feature-specific changes in memory representations (Hulbert & Norman, 2015; Norman et al., 2006; Norman et al., 2007).

As with the repulsion effects, the precision effects we observed are in sharp contrast to typical interference effects. Specifically, whereas prior studies have shown that interference *reduces* precision in feature memory (Berens et al., 2020; Pertzov et al., 2017; Sun et al., 2017), our findings reveal that memory interference was associated with a relative *gain* in memory precision when comparing the diagnostic versus non-diagnostic dimensions. Importantly, however, we defined precision as the standard deviation across repeated tests of the *same memory*. This measure of precision was orthogonal to repulsion (or accuracy) as it reflected the consistency with which faces were remembered, regardless of the distance between remembered and actual values (absolute error). Put another way, if each face feature is represented by a distribution of potentially remembered values, repulsion would reflect a *shift* in this distribution whereas precision would reflect reduced *variance* in this distribution (Yu & Geng, 2019). This is a key point because prior measures of memory precision have often assumed a distribution centered around the actual (veridical) memory value (e.g., Brady et al., 2013; Cooper & Ritchey, 2019; Harlow & Donaldson, 2013; Harlow & Yonelinas, 2016; Nilakantan et al., 2017; Nilakantan et al., 2018; Rhodes et al., 2020; Richter et al., 2016). While this is a reasonable assumption in many contexts, the current findings provide clear evidence, in the context of memory interference, that this assumption is violated.

An interesting avenue for future research will be to characterize the relationship between repulsion and precision. Here, these measures were mathematically distinct and were independently predictive of associative memory interference. Yet, repulsion and precision both have the consequence of increasing representational distance between competing memories, and may therefore serve a common purpose. In fact, there was a robust correlation between these measures, with greater repulsion predicting greater precision (Fig. S2A, OSM). Thus, it is possible that repulsion and precision are distinct facets of a common underlying mechanism.

In summary, we demonstrate that episodic memories are modified and distorted in targeted and adaptive ways in response to interference. Whereas it is intuitive to conceptualize interference resolution as a reduction in memory errors, our findings support a distinct view in which systematic memory errors enhance discriminability between similar memories.

## Supplementary information

ESM 1 (PDF 1768 kb)

## Data Availability

The datasets generated during and analyzed during the current study are available on the OSF project site (https://osf.io/ez2gs/).

## Code availability

Both experiment and analysis code are available on the OSF project site (https://osf.io/23s84/).
