# Supplementary material for: Long-term memory interference is resolved via repulsion and precision along diagnostic memory dimensions
Source: Psychon Bull Rev. 2022 Apr 5;29(5):1898–912. doi: 10.3758/s13423-022-02082-4 (PMC9568473; doi:10.3758/s13423-022-02082-4)
Supplement: Supplementary file 1 — (PDF 1768 kb) [file 13423_2022_2082_MOESM1_ESM.pdf]

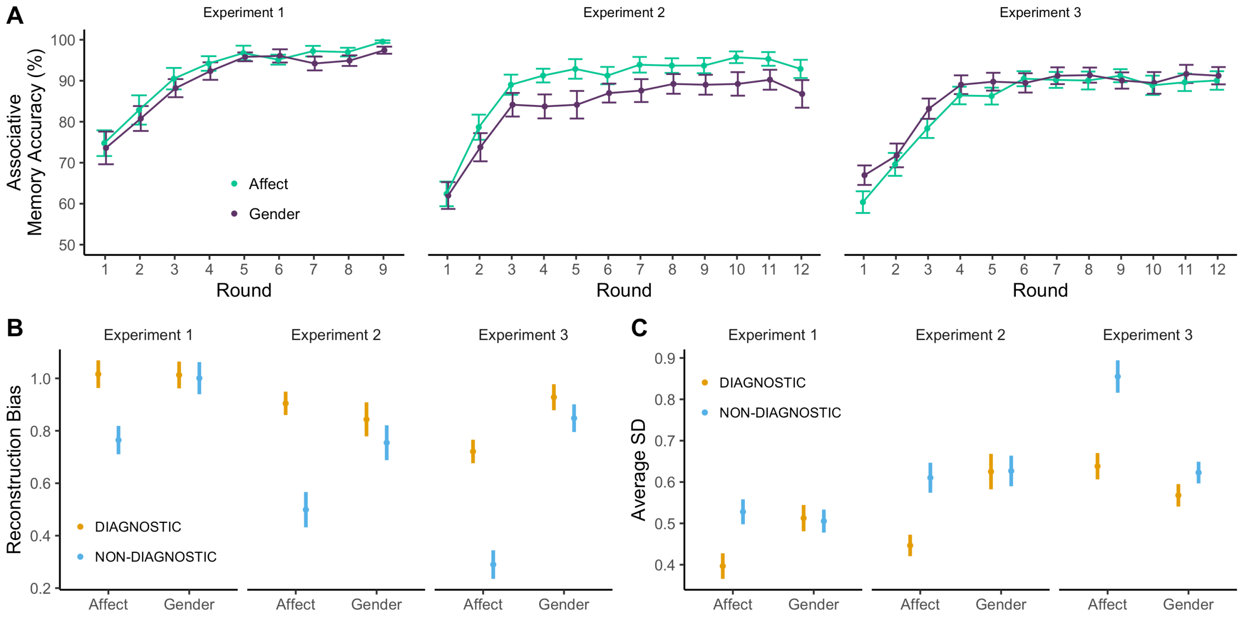

Fig. S1. Associative memory accuracy, reconstruction precision, and reconstruction bias according to whether the diagnostic dimension was affect vs. gender. **A.** Accuracy (percent correct) on the associative memory test during each round of the learning phase (competitive condition only) separated by the whether the diagnostic dimension was affect (green) or gender (purple) and by experiment number. For exp. 1, there was no difference in accuracy for affect vs. gender ($F(1,35) = 1.84$, $p = 0.18$, $\eta_G^2 = 0.004$). For exp. 2, the similarity between competitive pairmates was increased on both affect and gender (see Methods). Although not intended, this resulted in accuracy being significantly higher when affect was the diagnostic dimension compared to gender ($F(1,40) = 13.22$, $p < 0.001$, $\eta_G^2 = 0.026$). We addressed this difference in exp. 3 by further (and selectively) increasing the similarity between competitive pairmates along the affect dimension. This change was successful, as there was no longer a significant difference in accuracy when the diagnostic dimension was affect vs. gender ($F(1,56) = 2.22$, $p = 0.14$, $\eta_G^2 = 0.003$). **B.** Reconstruction bias as a function of dimension (diagnostic, non-diagnostic), whether the diagnostic dimension was affect or gender, and experiment number Here, bias was measured as the (un-modeled) mean response because there were too few trials to perform the modelling approach used in the main text. A repeated measures ANOVA revealed a robust main effect of dimension, reflecting significantly greater bias towards repulsion (higher mean reconstruction bias) on the diagnostic vs. non-diagnostic dimension ($F(1,118) = 85.05$, $p < 0.001$, $\eta_G^2 = 0.089$). However, there was also a significant interaction between diagnostic/non-diagnostic dimension and gender/affect ($F(1,118) = 34.41$, $p < 0.001$, $\eta_G^2 = 0.047$) reflecting a greater difference between diagnostic vs. non-diagnostic dimensions when the diagnostic dimension was affect (this effect did not further interact with experiment number, three-way interaction: $F(2,118) = 0.39$, $p = 0.68$, $\eta_G^2 = 0.001$). Nonetheless, the effect of diagnostic vs. non-diagnostic dimension was significant when the diagnostic dimension was affect ($F(1,118) = 88.44$, $p < 0.001$, $\eta_G^2 = 0.234$) or gender ($F(1,118) = 4.27$, $p = 0.041$, $\eta_G^2 = 0.008$). **C.** Reconstruction precision (SD of responses across the 4 reconstruction trials for each face) as a function of dimension (diagnostic, non-diagnostic), whether the diagnostic dimension was affect or gender, and experiment number. A repeated measures ANOVA revealed a robust main effect of dimension ($F(1,118) = 45.30$, $p < 0.001$, $\eta_G^2 = 0.054$), reflecting greater precision (lower SD) for the diagnostic than the non-diagnostic dimension. However, there was also a significant interaction between diagnostic/non-diagnostic dimension and gender/affect ($F(1,118) = 36.60$, $p < 0.001$, $\eta_G^2 = 0.034$), reflecting a greater difference between the diagnostic vs. non-diagnostic dimensions when the diagnostic dimension was affect (this effect did not further interact with experiment number, three-way interaction: $F(2,118) = 0.086$, $p = 0.92$, $\eta_G^2 < 0.001$). When affect was the diagnostic dimension, the effect of diagnostic vs. non-diagnostic dimension was significant ($F(1,118) = 76.34$, $p < 0.001$, $\eta_G^2 = 0.147$). In contrast, when gender was the diagnostic dimension, the effect of diagnostic vs. non-diagnostic dimension was not significant ($F(1,118) = 1.22$, $p = 0.27$, $\eta_G^2 = 0.003$). Note: error bars represent SEM.

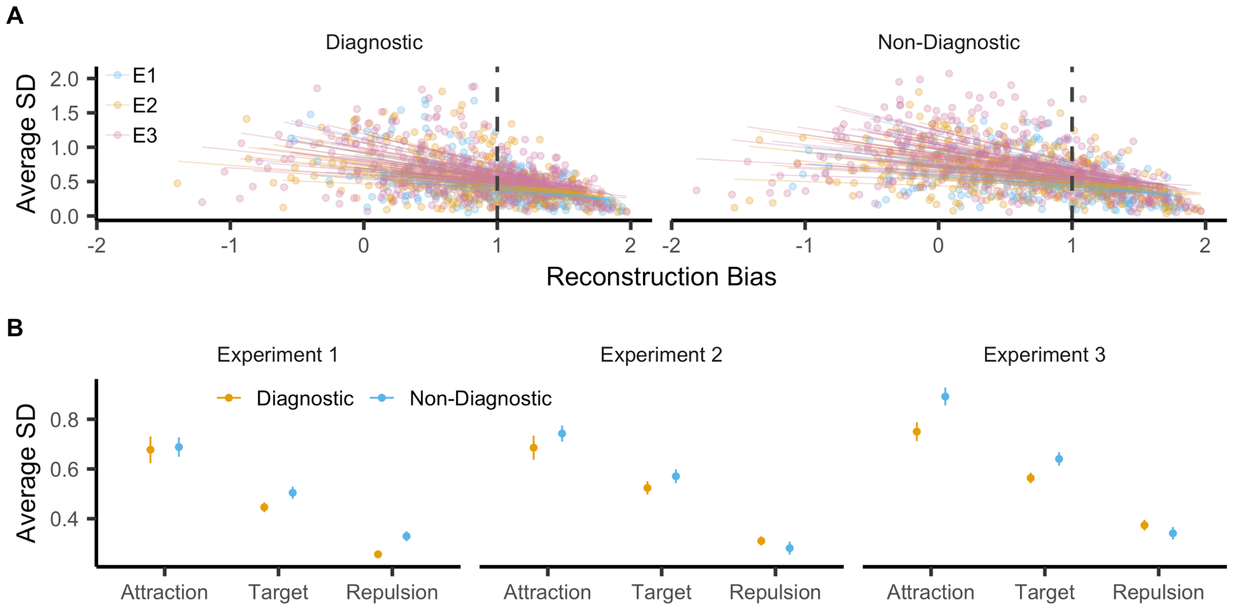

Fig. S2 Relationship between reconstruction bias and precision. **A.** A mixed-effects model was run with precision (mean SD) as the dependent variable and with experiment number (1, 2, 3), dimension (diagnostic, non-diagnostic) and bias included as predictors. Measures of precision and bias were computed at the item level, with each measure based on the mean value across the 4 reconstruction trials for each face. The relationship between bias and precision was modeled with random intercepts and slopes for each participant. Compared against a model without bias, adding bias significantly improved model fit ($\chi^2(1) = 140.8$, $p < 0.001$, $\beta_{bias} = -0.25$, $SE = 0.015$) reflecting the fact that stronger bias (repulsion) was associated with greater precision (lower SD). In the plot, each dot represents a specific face image, each experiment is a unique color (e1: blue; e2: orange; e3: pink), and each line represents the modelled, participant-specific relationship between reconstruction bias and precision. Notably, the effect of bias on precision did not interact with dimension type (diagnostic vs. non-diagnostic: $\chi^2(1) = 1.61$, $p = 0.20$, $\beta_{biasXdim} = 0.027$, $SE = 0.022$, or experiment: $\chi^2(2) = 2.32$, $p = 0.31$). Thus, although the diagnostic dimension was associated with greater bias and precision compared to the non-diagnostic dimension (see main text), the relationship between bias and precision was not specific to the diagnostic dimension. **B.** One potential account of why precision was greater on the diagnostic dimension is that greater repulsion (towards the boundary) reduced the response space for reconstruction (compressing variance). To address this, we computed mean precision for each dimension as a function of the level of bias on the diagnostic and non-diagnostic dimensions. Three equal-width bias bins were created within the half of the response range that was closer to the target than the competitor (0—2): 'Attraction' represents bias in the direction of the competitor face (range of bias values = 0–0.67); 'Target' represents responses centered around the true value (range = 0.67–1.33); 'Repulsion' represents bias away from the competitor face (range = 1.33–2). Because not all participants contributed to each bin, the mean and SEM were calculated across items, ignoring participant. Qualitatively, while precision was markedly higher, overall, for the Repulsion bin (lower SD), the tendency for greater precision on the diagnostic vs. non-diagnostic dimension was *not* selective to the Repulsion bin—in fact, the effect was least consistent in the Repulsion bin. In order to statistically confirm that the difference in precision for diagnostic vs. non-diagnostic dimensions was not an artifact of high bias values, we calculated the mean precision for the diagnostic and non-diagnostic dimensions only including faces within the Attraction and Target bins (0–1.33). Data were included from all experiments, but one participant (from e3) was excluded for having no items within the specified range. The remaining participants each had at least 2 items in the specified range for both the diagnostic ($M = 5.70$) and non-diagnostic ($M = 5.16$) dimensions (out of 8 possible items). Even with this restricted range (that excluded high bias items), there was significantly greater precision on the diagnostic compared to the non-diagnostic dimension ($F(1,117) = 25.16$, $p < 0.001$, $\eta^2_G = 0.051$). This effect did not interact with experiment ($F(2,117) = 2.20$, $p = 0.12$, $\eta^2_G = 0.009$). Note: error bars represent SEM.

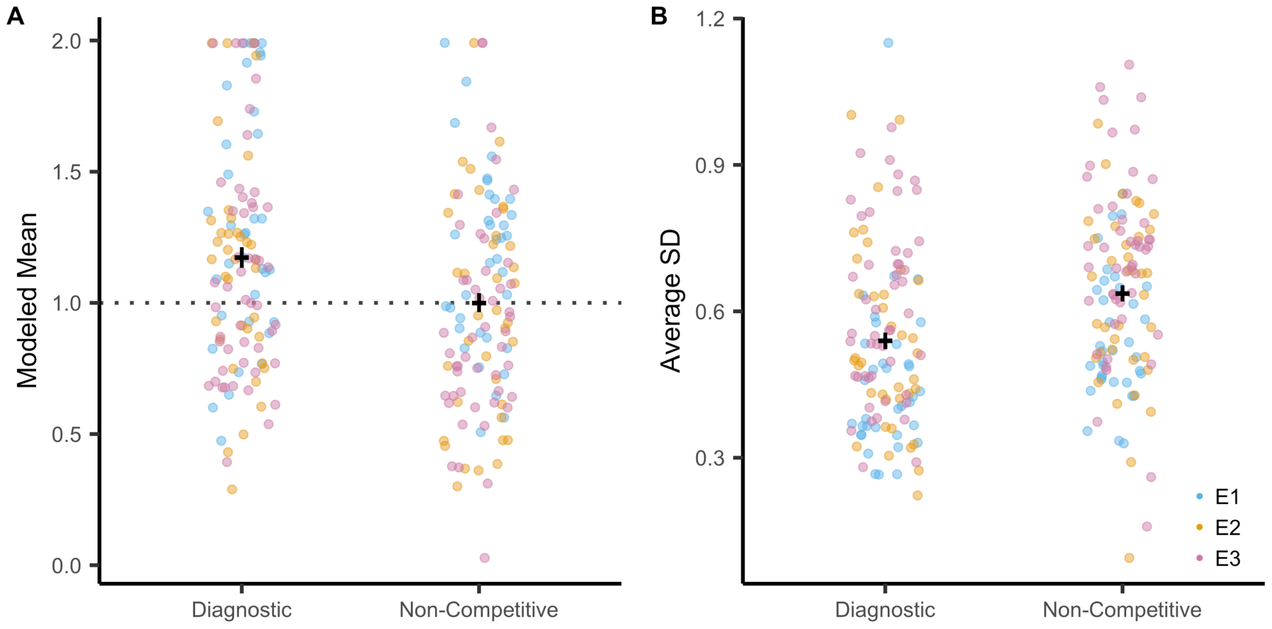

Fig. S3 Reconstruction bias and precision for faces in the competitive vs. non-competitive conditions. For the non-competitive condition there was no distinction between diagnostic vs. non-diagnostic dimensions. Thus, for each face in the non-competitive condition, data from both dimensions were included. With 4 items in the non-competitive condition for each participant, and 4 reconstruction trials per face, this yielded 32 total values per participant for the non-competitive condition (2 dimensions x 4 items x 4 trials). Bias was modeled using the same method as for the diagnostic and non-diagnostic dimensions (see Methods). **A.** Bias was significantly greater (higher modeled mean) for the diagnostic dimension (of faces in the competitive condition) than for the non-competitive condition ($F(1,118) = 22.11$, $p < 0.001$, $\eta_G^2 = 0.043$). This difference did not interact with experiment ($F(2,118) = 0.13$, $p = 0.88$, $\eta_G^2 < 0.001$). There was also a significant difference between the non-diagnostic dimension and the non-competitive condition ($F(1,118) = 6.73$, $p = 0.011$, $\eta_G^2 = 0.015$; not shown in the figure), with no interaction by experiment ($F(2,118) = 1.90$, $p = 0.15$, $\eta_G^2 = 0.008$). Specifically, for the non-diagnostic dimension there was a relative bias toward the center of face space (modeled mean tending to be lower than 1; see **Figure 4**) whereas for the non-competitive condition the modeled mean was higher (almost exactly at the true value of 1). **B.** Precision was significantly greater (lower mean SD) for the diagnostic dimension (of faces in the competitive condition) than for the non-competitive condition ($F(1,118) = 39.44$, $p < 0.001$, $\eta_G^2 = 0.073$). This difference did not interact with experiment ($F(2,118) = 0.12$, $p = 0.89$, $\eta_G^2 < 0.001$). Notably, there was no significant difference in precision between the non-competitive condition and the non-diagnostic dimension ($F(1,118) = 0.006$, $p = 0.94$, $\eta_G^2 < 0.001$; not shown in the figure), nor was there an interaction by experiment ($F(2,118) = 1.75$, $p = 0.18$, $\eta_G^2 = 0.006$). Notes: Each dot represents a participant, with color indicating the experiment (e1: blue; e2: orange; e3: pink); error bars represent SEM.

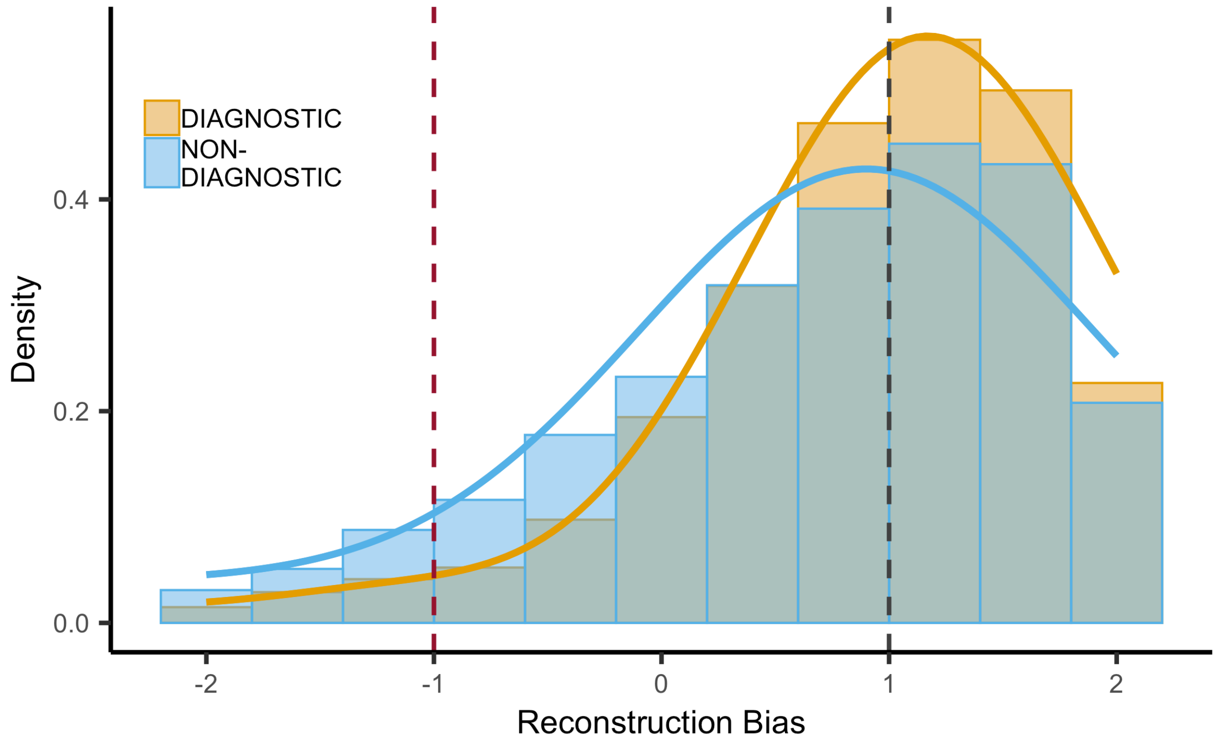

Fig. S4 Histogram of reconstruction responses across all experiments, participants, and items in the competitive condition. Responses were separated by whether the dimension was diagnostic (orange) or non-diagnostic (blue). As in all other analyses, responses were rescaled such that the location of the target was at 1 (black dotted line), the center of the face space was at 0, and in the case of the diagnostic dimension, the location of the competitor was at -1 (red dotted line). To better characterize the distributions, separate mixture models were generated for the diagnostic and non-diagnostic dimensions. Each model included three distributions: a target distribution (the correct face), a competitor distribution (the competitor face), and a uniform distribution (random guessing). For the target distribution, we used a truncated normal distribution where we set the mean to the mean of our estimate from the main bias analysis across all participants from all experiments (diagnostic: 1.17; non-diagnostic: .9) and allowed the standard deviation to vary within a 'generous' range that was wide beyond what would plausibly explain the data (0.3–2). We used the same approach for the competitor distribution but changed the mean. For the diagnostic dimension, we mirrored the target bias value by setting the competitor value to -1.17. For the non-diagnostic dimension, since there was no competitor, we set the competitor value at the value where a competitor would be (-1). Although there was no competitor in the case of the non-diagnostic dimension, we included it here to allow a fairer comparison across the diagnostic and non-diagnostic dimensions. In particular, the non-diagnostic dimension allows for a baseline estimate of the percentage of swap errors (recalling the competitor) in a situation where there should not be any. For the diagnostic dimension, the best fitting model estimated that the target distribution explained 91.9% of responses (SD = .8), as reflected by the orange line. The model estimated that 6.1% of responses were random guesses and 2.0% of responses were swap errors (SD = .5). For the non-diagnostic dimension, the best fitting model estimated that the target distribution explained 84.1% of responses (SD = 1), as reflected by the blue line. The model estimated that 15.9% of responses were random guesses and 0% were swap errors. Taken together, these mixture model results suggest that the target distributions largely explained responses, with relatively little influence from random guesses and swap errors. That said, because the mixture models require a relatively high number of data points, these models were not well-suited to characterizing distributions for individual items (faces) and participants.
